# Realization of collimated specific profiles in rotation-symmetrical beam shaping system with divergent light source

**DOI:** 10.1186/s11671-023-03934-1

**Published:** 2023-12-21

**Authors:** Cheng-Mu Tsai, Tzu-Chen Yu, Pin Han, Yi-Chin Fang

**Affiliations:** 1 Graduate Institute of Precision Engineering, National Chung Hsing University, Taichung City, 402 Taiwan; 2 Institute of Smart Industry and Green Energy, National Yang Ming Chiao Tung University, Tainan City, 711 Taiwan

**Keywords:** Beam shaping, Aspherical surface, Non-imaging optics, Collimation

## Abstract

A simple numerical method is proposed for the design of two aspherical surfaces, each comprising multiple segmented refractive planes, for generating a collimated beam with a specific irradiance profile in a beam shaping system with a divergent light source. However, in real-world manufacturing, this performance improvement is obtained at the expense of a greater cost and complexity. Accordingly, a second algorithm is proposed which maximizes the number of rays passing through the central regions of the refractive planes in the second aspherical surface and hence minimizes the total number of segments required to achieve the same beam shaping performance. The feasibility of the proposed method is demonstrated through the design of two aspherical lenses for generating collimated output beams with ring- and triangle-like irradiance profiles, respectively. The experimental results show that the beam profiles are in close agreement with the desired irradiance distributions. In general, the results indicate that the proposed method provides a versatile and efficient approach for achieving the desired collimated profile in beam forming systems with a divergent light source.

## Introduction

Many optical systems nowadays require light sources to be pre-shaped into desired light distributions. Thus, many beam shaping methods have been proposed [1–24]. Most of these studies have focused on the problem of achieving a uniform light distribution [3–7]. For example, Cao et al. [5] proposed a beam shaping system which produced an output beam with a uniform light distribution using two micro-lens arrays and a second lens. Wang et al. [6] proposed a 4*f* system architecture consisting of two spatial light modulators and two positive lenses with a focal length of *f* to achieve a uniform light profile. The main objective of the beam shaping systems in [5, 6] is simply to achieve a uniform light distribution as the output. However, in some applications, such as light detection and ranging (LiDAR) [25], the light distribution must not only be a specific profile, but also collimated.

The problem of achieving collimated uniform light was first addressed as early as 1965 by Frieden [8], who utilized two refractive aspherical lenses to achieve a collimated uniform light output with no power loss. However, there are some flaws in Frieden's [8] design for achieving collimated uniform distribution because the two surfaces of the first and second aspherical lenses are interdependent. Thus, when adjusting the structure of one surface, the surface of the other lens must also be modified in order to maintain the same output. Rhodes and Shealy [9] addressed this problem by actively considering the interdependence of the two aspherical surfaces in the design process and developing relevant formulae for the two surfaces accordingly. In a more recent study, Liu and Zhang [16] reviewed existing methods for collimated beam shaping systems and proposed several alternative lens structures. However, all of the methods in [8–18] are designed specifically for optical systems with a collimated light source.

Some studies [19–23] presented several methods for the beam shaping of divergent light sources. However, these methods do not specifically focus on the simultaneous collimation and formation of specific light profile outputs. The integrable ray mapping method [24] could be a way to design two freeform surfaces catering to specific collimated light distributions. As an alternative, a numerical method [12, 13, 18] is proposed for easier construction of the aspherical lens. The simulation results confirm that the designed lenses fulfill the twin requirements of collimation and uniform light distribution, respectively. However, in real-world lens systems, the cost and complexity of the lens manufacturing process scale with the number of refractive planes. Previous studies have shown that when the reference rays pass through the central region of a refractive surface, the same beam shaping performance can be achieved using fewer refractive plane segments [12, 13]. As yet, there are no direct formulae available to ensure that the reference beam passes through the center region of the second aspherical refractive plane of aspherical lenses. Thus, the present study proposes a novel algorithm that maximizes the passage of the reference beams through the center regions of the related refractive planes in the second aspherical surface.

The simulation results demonstrate that the lens designed with the proposed algorithm achieves the same beam shaping performance with fewer segments of the refractive plane than the original lens. Furthermore, by evaluating the power distribution of the divergent light source on the first aspherical surface, the algorithm can be used to design the second aspherical surface in such a way as to generate a specific irradiance profile of the collimated output beam. The feasibility of the proposed approach is demonstrated using two light profile outputs for illustration purposes, namely ring-like and triangle-like. The simulation and experimental results confirm that the proposed method achieves the overall goal of producing collimated specific light profile outputs.

The remainder of this paper is organized as follows. Section 2 introduces the fundamental aspherical lens design method employed to obtain a collimated uniform beam profile. Section 3 describes the algorithm used to minimize the number of refractive segments of the second aspherical lens. Section 4 presents and discusses the simulation and experimental results for the two considered light profiles. Finally, Sect. 5 provides some brief concluding remarks.

## Fundamental aspherical lens design for collimated uniform beam profile

**Fig. 1:**
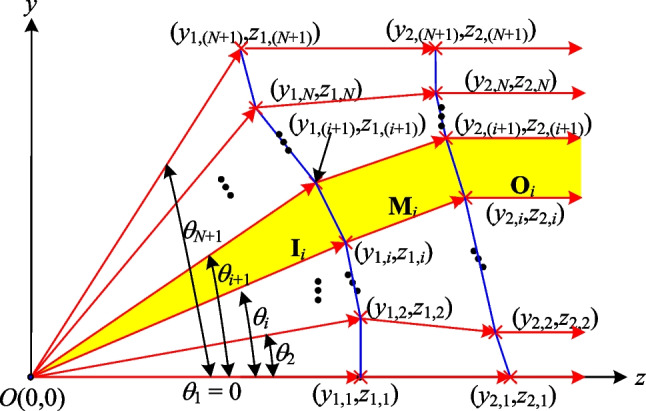
Aspherical lens design for divergent rotationally symmetric light source

To achieve both uniform and collimated light distribution, it is necessary to use at least two aspherical surfaces, where the first surface is responsible for evenly distributing the power onto the second aspherical surface, while the second lens handles the collimation process (see Fig. 1). It is important to note that the two aspherical surfaces are not independent of one another but rather mutually affect each other. That is, changing the structure of one surface inevitably affects the structure of the other. Therefore, both surfaces must be considered simultaneously during the design process.

For the case considered in the present study of a beam forming system with a divergent light source, each of the two aspherical surfaces is assumed to be divided into *N* segments in order to achieve a uniform distribution of the output power. Furthermore, each segment is assumed to carry an equal amount of power to meet the need for uniformity of the output light. Since the light source is divergent and rotationally symmetric, the angular intervals corresponding to the same amount of power can be denoted simply as (*θ*_1_, *θ*_2_, …, *θ*_*N*+1_), where *N* is the number of segments. Furthermore, the corresponding heights of the segments on the first aspherical surface can be denoted as (*y*_1,1_,* y*_1,2_, …, *y*_1,(*N*+1)_). Theoretically, a uniform distribution of the output light pattern can be obtained by designing the second aspherical surface such that the same power is distributed over intervals of equal area heights (*y*_2,1_,* y*_2,2_, …, *y*_2,(*N*+1)_), as shown in Fig. 1.

Without loss of generality, let the light source be a point source with a rotationally symmetric divergent Gaussian distribution. For a point source with rotational symmetry, the original two-dimensional light distribution can be reduced to a single dimension for convenience in designing the aspherical lens. The power distribution of a rotationally symmetric divergent Gaussian beam is given as follows:

1 $$I\left(\theta \right)={I}_{0}{e}^{{-2(\theta /\sigma )}^{2}},$$

where *I*_0_ is the radiation power intensity at the center, and σ is a parameter representing the divergence angle. As described above, for the problem considered in the present study, the power of the incident light source is to be the same for each segment of the first aspherical surface. Since the divergent light source is rotationally symmetric, the angles that yield the same power are given as

2 $$\begin{array}{ccc}\begin{array}{l}{P}_{1,i}={\int }_{{\theta }_{i}}^{{\theta }_{i+1}}{I}_{0}{e}^{{-\left(\frac{\theta }{\sigma }\right)}^{2}}sin\theta d\theta \\ \quad \quad=\frac{{\int }_{{\theta }_{1}=0}^{{\theta }_{N+1}}{I}_{0}{e}^{{-\left(\frac{\theta }{\sigma }\right)}^{2}}sin\theta d\theta }{N}={\text{const}}\end{array}& \mathrm{for}& i = 1, 2, \dots , N,\end{array}$$

where *θ*_*N*+1_ represents the maximum divergent angle of the light source, and *θ*_1_ = 0. The corresponding angles can be determined using a numerical method. When the rays from the equal power angle segments, as determined by solving Eq. (2), are refracted to the corresponding height segments on the second aspherical surface with the same area, the second aspherical surface produces a uniform distribution of the output light. The heights of the segments on the second surface which satisfy this condition can be determined by solving the equation

3 $$\begin{array}{*{20}c} {y_{{{2},i}} = \left[ {\left( {i - {1}} \right)R_{{2}}^{{2}} /N} \right]^{{{1}/{2}}} } & {{\text{for}}} & { i = 1,\;2,\, \ldots ,N + 1,} \\ \end{array}$$

where *R*_2_ is the radius of the output beam.

**Fig. 2:**
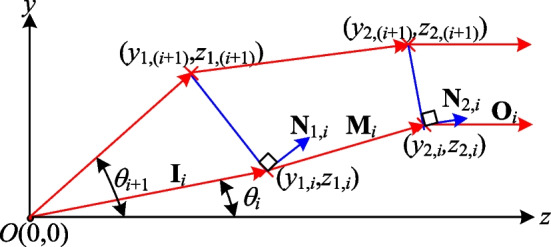
Proposed construction method for surface profiles of two aspherical surfaces given divergent rotationally symmetric light source

Figure 2 illustrates the process employed in the present study to construct the segmented refractive planes (represented by the blue line segments) of two aspherical surfaces which collectively achieve a uniform light output with a transformation from a rotationally symmetric diverging light source to a rotationally symmetric collimated light output with a specified profile. Assume that the coordinates of two related points (*y*_1,*i*_, *z*_1,*i*_) and (*y*_2,*i*_, *z*_2,*i*_) on the first and second aspherical surfaces, respectively, are known. The coordinates of these points can be used to determine the refracted beam vector **M**_*i*_ such that the input beam, after refraction at point (*y*_1,*i*_, *z*_1,*i*_) on the first aspherical surface, reaches point (*y*_2,*i*_, *z*_2,*i*_) on the second aspherical surface and achieves a uniform distribution of the output light. The required refractive vector can be determined mathematically as

4 $$M_{i} = \frac{{\left[ {0 \left( {y_{2,i} - y_{1,i} } \right) \left( {z_{2,i} - z_{1,i} } \right)} \right]}}{{\sqrt {\left( {y_{2,i} - y_{1,i} } \right)^{2} + \left( {z_{2,i} - z_{1,i} } \right)^{2} } }}$$

The normal vector of the *i*-th segmented refractive plane on the first aspherical surface is denoted as **N**_1,*i*_ = [0 *n*_(1,1),*i*_* n*_(1,2),*i*_] and can be obtained simply from Snell's law as

5 $$\left[ {{1} + n_{r}^{{2}} {-}{ 2}n_{r} \left( {{\mathbf{M}}_{i} \cdot {\mathbf{I}}_{i} } \right)} \right]^{{{1}/{2}}} {\mathbf{N}}_{{{1},i}} = {\mathbf{M}}_{i} {-}n_{r} {\mathbf{I}}_{i,}$$

where *n*_*r*_ = 1/*n*, in which *n* is the refractive index of the material used to manufacture the aspherical lens, and **I**_*i*_ is the unit vector of the incident light on the *i*-th segment. Due to the divergent nature of the light source, the incident vector **I**_*i*_ can be obtained as [0 sin*θ*_*i*_ cos*θ*_*i*_]. Having determined the incident vector **I**_*i*_ and refracted vector **M**_*i*_, Snell's law can be used to calculate the required normal vector **N**_1,*i*_ = [0 *n*_(1,1),*i*_* n*_(1,2),*i*_] for the *i*-th segmented refractive plane on the first aspherical surface.

Given the point (*y*_1,*i*_, *z*_1,*i*_) on the first surface, it is necessary to calculate the point (*y*_(1,*i*+1)_, *z*_(1,*i*+1)_) on the same plane in order to construct the corresponding segmented refractive plane. This point can be determined by considering the intersection of two lines, namely one line which represents the light ray with the next incident angle (*θ*_*i*+1_) and is given mathematically as

6 $$y = z\tan (q_{i + 1} ),$$

and a second line which represents the segmented refractive plane of the first aspherical surface and has the equation

7 $$y = mz + b,$$

where *m* =  − *n*_(1,2),*i*_/*n*_(1,1),*i*_ and *b* = *y*_1,*i*_ − *mz*_1,*i*_ (see Fig. 2).

Solving Eqs. (6) and (7) simultaneously yields the point (*y*_(1,*i*+1)_, *z*_(1,*i*+1)_), which marks the end of first segment of the first aspherical surface. When the incident light strikes point (*y*_1,*i*_, *z*_1,*i*_) on the aspherical surface with an incident angle of *θ*_*i*_, it is refracted by the segmented refractive plane formed by points (*y*_1,*i*_, *z*_1,*i*_) and (*y*_(1,*i*+1)_, *z*_(1,*i*+1)_) such that it reaches point (*y*_2,*i*_, *z*_2,*i*_) on the second aspherical surface and achieves a uniform light distribution.

Adopting the design approach outlined above for each segment of the first aspherical surface, the divergent light source, after refraction, has a uniform distribution on the second aspherical surface. However, to meet the overall goal of the present study, namely that of achieving both uniformity and collimation, the second aspherical surface must be designed such this uniformly distributed beam is output in a collimated manner. Since a uniform distribution is achieved when the same amount of power is distributed over intervals of equal area heights on the second aspherical surface (see Fig. 1), the heights of the segments on the second surface are already known as (*y*_2,1_, *y*_2,2_, …, *y*_2,*N*+1_). Thus, to construct the second aspherical surface, it is necessary only to determine the coordinate of each point in the *z*-direction, i.e., *z*_2,(*i*+1)_, for each segmented refractive plane. Assume that **M**_*i*_ represents the incident vector on the second aspherical surface and the output vector is to have the form of collimated light with **O**_*i*_ = [0 0 1]. As for the construction process described above for the first aspherical surface, it is necessary to calculate the normal vector of each segment on the second surface at point (*y*_2,*i*_, *z*_2,*i*_). By applying Snell's law once again, the normal vectors **N**_2,*i*_ = [0 *n*_(2,1),*i*_* n*_(2,2),*i*_] can be obtained as

8 $$\left[ {{1} + n^{{2}} {-}{2}n\left( {{\mathbf{O}}_{i} \cdot {\mathbf{M}}_{i} } \right)} \right]^{{{1}/{2}}} {\mathbf{N}}_{{{2},i}} = {\mathbf{O}}_{i} {-}n{\mathbf{M}}_{i} .$$

By considering the geometric relationship between points (*y*_2, (*i*+1)_, *z*_2, (*i*+1)_) and (*y*_2,*i*_, *z*_2,*i*_), point *z*_2, (*i*+1)_ can be determined as

9 $${z}_{2,(i+1)}={z}_{2,i}-\frac{{n}_{\left(\mathrm{2,1}\right),i}}{{n}_{\left(\mathrm{2,2}\right),i}}({y}_{2,\left(i+1\right)}-{y}_{2,i}).$$

The method described above gives the points (*y*_1,(*i*+1)_, *z*_1,(*i*+1)_) and (*y*_2,(*i*+1)_, *z*_2,(*i*+1)_) on the first and second aspherical surfaces, respectively. By applying the method recursively, all of the segments on the two surfaces can be obtained such that a uniform and collimated light output is achieved. The initial points of the two aspherical surfaces lie on the same horizontal axis, specifically (*y*_1,1_, *z*_1,1_) = (0, *d*) and (*y*_2,1_, *z*_2,1_) = (0, *d* + *t*), respectively, where *d* is the distance between the light source and the first aspherical lens and *t* is the thickness of the lens. The surfaces of the lens can be obtained numerically using Eqs. (2)–(9), where the first surface yields a uniform light distribution on the second surface, and the second surface produces a uniform and collimated light output.

**Fig. 3:**
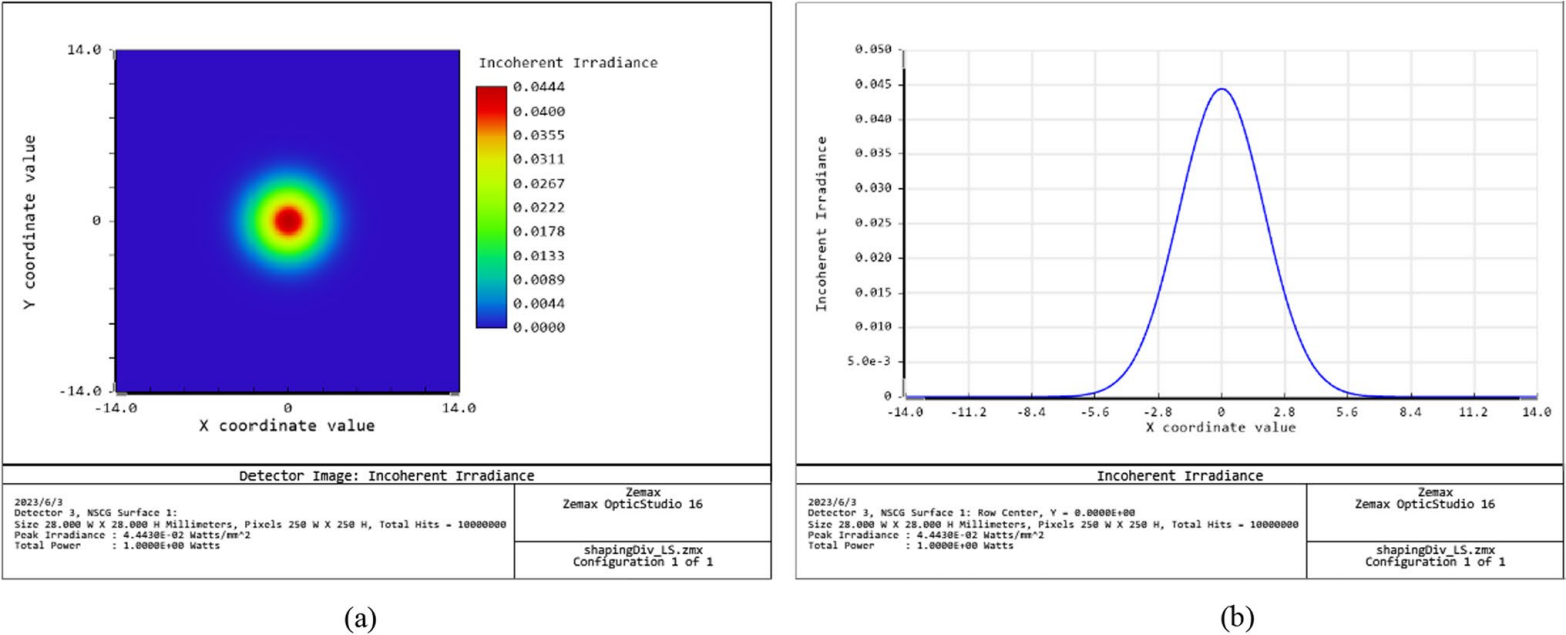
Zemax OpticStudio simulation results for profile of considered light source: **a** 2D spot irradiance, and **b** cross-section of spot irradiance

The feasibility of the proposed designed method was evaluated using the light source shown in Fig. 3, in which the light source had a divergent Gaussian profile with a maximum divergent angle of 16 degrees and the detector was located at a distance of *d* = 25 mm from the light source. The simulations considered the design of a simple aspherical lens consisting of just *N* = 5 segments for each surface. The lens was assumed to be fabricated of PMMA and to have a thickness of 35 mm. The aim of the designed lens was to produce a collimated uniform output beam spot with a radius of 11.3 mm.

**Fig. 4:**
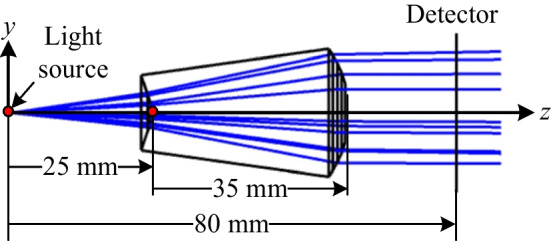
2D layout of designed aspherical lens for simple case of *N* = 5

**Fig. 5:**
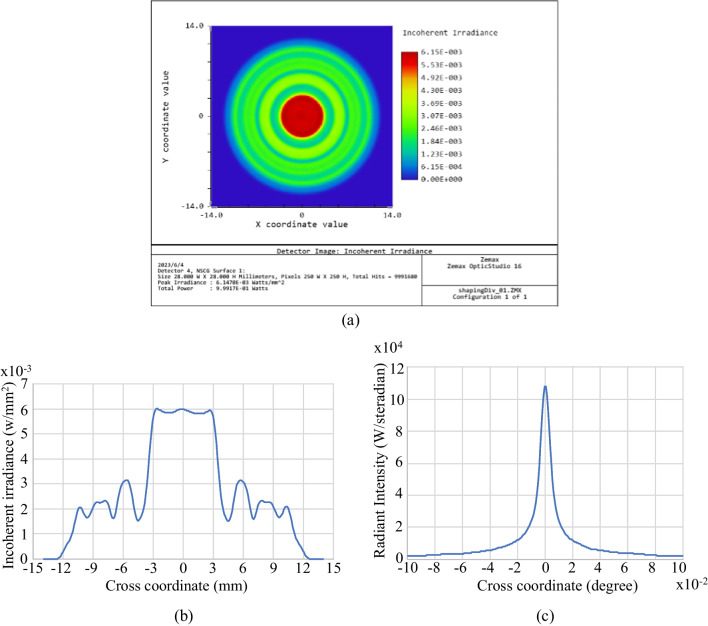
Simulation results for aspherical lens with *N* = 5: **a** 2D spot irradiance, **b** cross-section of spot irradiance, and **c** cross-section of radiance intensity

**Fig. 6:**
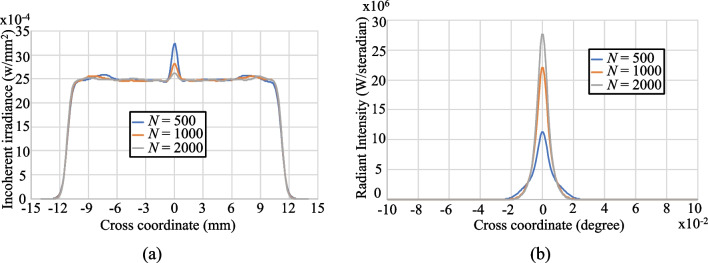
Simulation results for aspherical lenses with *N* = 500, 1000, and 2000 segments: **a** cross-sections of spot irradiance and **b** cross-sections of radiance intensity

Table 1 shows the results obtained from Eqs. (2)~(9) for the point coordinates of the two aspherical surfaces. Figure 4 shows the corresponding layout of the aspherical lens, where the light source is located 25 mm in front of the lens and the detector is placed 80 mm behind the light source. There are 10 millions of rays used in the simulation. The detector is set to 250 × 250 pixels. The results presented in Fig. 5a and b show that the output beam has a relatively poor distribution uniformity since the aspherical surfaces consist of just *N* = 5 segments. Nonetheless, the radiant intensity results presented in Fig. 5c show that the lens achieves a good collimation performance, with a divergence of just ± 0.02 degrees. Figure 6a and b show the results obtained when increasing the number of segments to 500, 1000, and 2000, respectively. The output profile becomes increasingly uniform as the number of segments increases, and the lens maintains an excellent collimation performance of around ± 0.02 degrees.

**Table 1:** Aspherical point coordinates for *N* = 5

Pair #	1	2	3	4	5	6
Surface 1	(0,25)	(1.254,25)	(1.888,24.855)	(2.512,24.659)	(3.299,24.377)	(6.654,23.206)
Surface 2	(0,60)	(5.054,60)	(7.147,59.301)	(8.753,58.537)	(10.107,57.751)	(11.3, 56.979)

## Aspherical lens design based on sparse segmented refractive planes

**Fig. 7:**
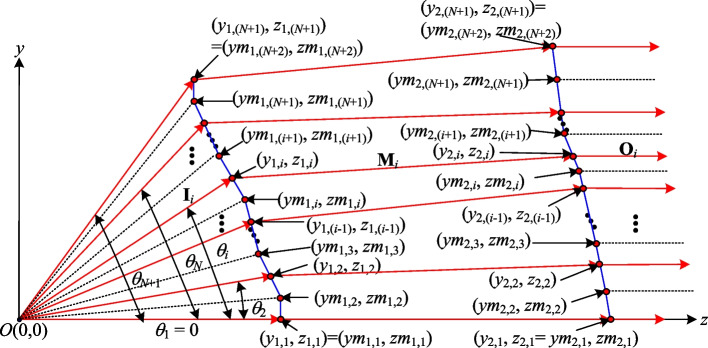
Aspherical lens design based on centers of segmented refractive planes

Previous studies [12, 13] have shown that when the incident ray **I**_*i*_, which serves as the reference ray, is directed towards the center of the segmented refractive plane, it produces a superior light pattern, and thus fewer segments of the refractive plane are required to construct the aspherical lens. Accordingly, a second algorithm was proposed to improve the beam shaping performance by constraining the reference beam **I**_*i*_ to pass as close as possible through the center of each designed refractive plane on the second aspherical surface. Figure 7 illustrates the proposed construction process for the two new aspherical surfaces. Initially, Eqs. (2) and (3) are employed to calculate the angles (*θ*_1_, *θ*_2_, …, *θ*_*N*+1_) which yield the same power on the first surface and the heights (*y*_2,1_, *y*_2,2_, …, *y*_2,*N*+1_) on the second surface, respectively. The average values of the adjacent angles of the first surface and the midpoints of the heights between the adjacent points on the second surface are then computed respectively as

10a $$\begin{array}{*{20}c} {qm_{i} = (q_{i - 1} + q_{i} )/2} & {{\text{for}}} & {i = 2, \ldots ,N + 1} \\ \end{array}$$

10b $$\begin{array}{*{20}c} {ym_{{{2},i}} = \left( {y_{{{2},i - {1}}} + y_{{{2},i}} } \right)/{2}} & {{\text{for}}} & {i = {2}, \ldots ,N + {1}} \\ \end{array}$$

where *θm*_1_ = *θ*_1_, *ym*_2,1_ = *y*_2,1_ and *ym*_2,*N*+2_ = *y*_2,*N*+1_.

**Fig. 8:**
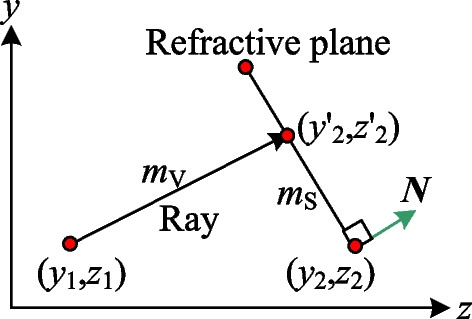
Relationship between incident ray and refractive plane

In illustrating the algorithm proposed in this study for constructing an aspherical surface that causes the reference beam **I**_*i*_ to pass through the center of the segmented refractive plane, it is necessary to first explain the method used to obtain the position coordinates of any general beam passing through the refractive plane. Referring to Fig. 8, points (*y*_1_, *z*_1_) and (*y*_2_, *z*_2_) are known, and the normal vector **N** = [0 *n*_1_ *n*_2_] of the refractive plane is also known. Given the direction cosine of the beam's propagation direction, the slope, *m*_V_, of the propagating ray can be easily determined from geometric principles. Moreover, the slope of the refractive surface, *m*_S_, can be calculated as − *n*_2_/*n*_1_. Thus, the coordinates of point (*y*_2_', *z*_2_') at which the beam and refractive surface intersect can be obtained as follows:

11 $$\left[\begin{array}{cc}1& -{m}_{V}\\ 1& -{m}_{S}\end{array}\right]\left[\begin{array}{c}{y^{\prime}}_{2}\\ {z^{\prime}}_{2}\end{array}\right]=\left[\begin{array}{c}{y}_{1}-{m}_{V}{z}_{1}\\ {y}_{2}-{m}_{S}{z}_{2}\end{array}\right].$$

**Fig. 9:**
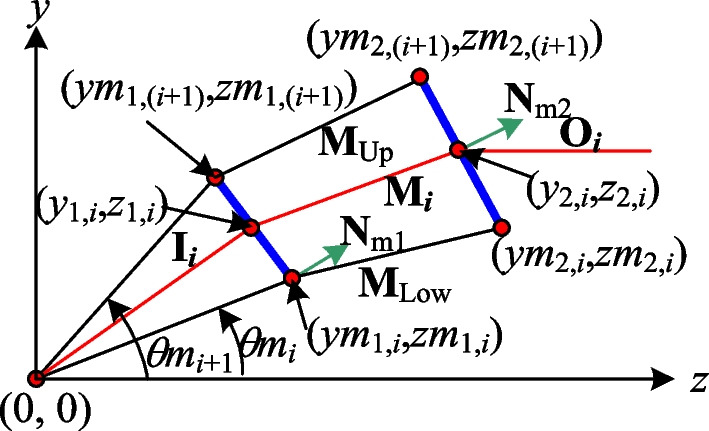
Construction method for refractive plane based on center points

**Fig. 10:**
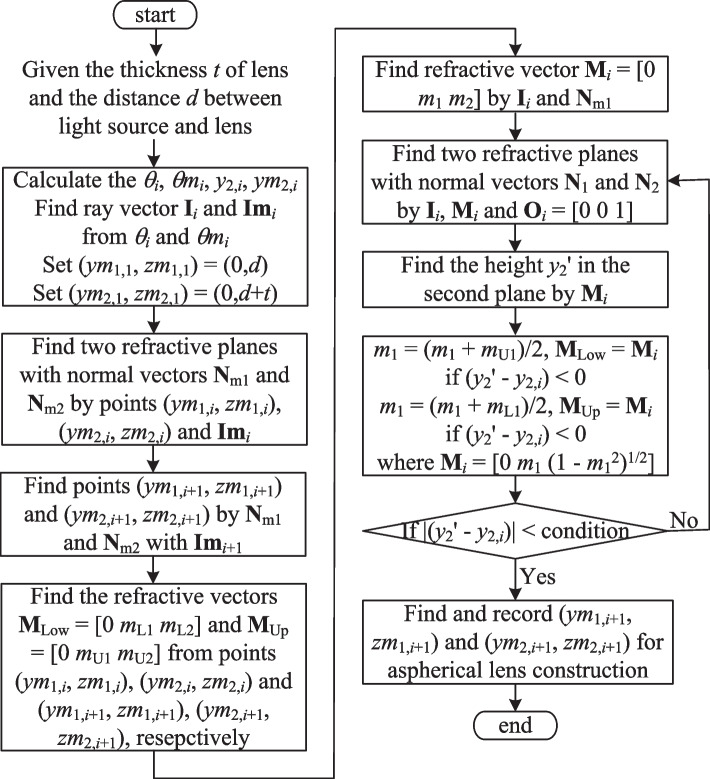
The algorithm flow chart for sparse segments

Since there are no direct formulae available for ensuring that the reference beam **I**_*i*_ passes through the center of the refractive surface in a beam forming system with a divergent light source, a numerical approach is required. The present study proposes an alternative algorithm in which the related refractive planes of the two aspherical surfaces are continuously adjusted until the solution is found which causes the reference beam **I**_*i*_ to pass through the center of the refractive plane. The center of the refractive plane is half the sum of the two boundary points, i.e., [(*ym*_2,*i*_, *zm*_2,*i*_) + (*ym*_2,*i*+1_, *zm*_2,*i*+1_)]/2. Therefore, when the reference beam **I**_*i*_ passing through the height [*ym*_2,*i*_ + *ym*_2,*i*+1_]/2 in the second refractive plane, we consider that the second refractive plane is corresponding the requirement for aspherical lens construction. Referring to Fig. 9, the main steps in the proposed algorithm are as follows:

- *Step 1* Points (*ym*_1,*i*_, *zm*_1,*i*_) and (*ym*_2,*i*_, *zm*_2,*i*_) on the two aspherical surfaces are defined and are used to construct two refractive planes with normal vectors **N**_m1_ and **N**_m2_, respectively. The refractive vector constructed from points (*ym*_1,*i*_, *zm*_1,*i*_) and (*ym*_2,*i*_, *zm*_2,*i*_) is denoted as **M**_Low_ = [0 *m*_L1_ *m*_L2_]. Similarly, the refractive vector constructed from points (*ym*_1,*i*+1_, *zm*_1,*i*+1_) and (*ym*_2,*i*+1_, *zm*_2,*i*+1_), obtained from a beam with an incident angle of *θm*_*i*+1_, is denoted as **M**_Up_ = [0 *m*_U1_ *m*_U2_]. However, these initial refractive planes do not guarantee that the reference beams pass through the centers of the corresponding planes of the second surface. Consequently, further processing is required.
- *Step 2* The refractive vector **M**_*i*_ = [0 *m*_1_ *m*_2_] generated when the reference beam **I**_*i*_ passes through the first refractive plane is computed and used to calculate the height *y*_2_' at which this refractive vector passes through the second refractive plane.
- *Step 3* When (*y*_2_' − *y*_2,*i*_) < 0, *m*_1_ is set as *m*_1_ = (*m*_1_ + *m*_U1_)/2, **M**_Low_ is updated as **M**_*i*_. Conversely, when (*y*_2_' − *y*_2,*i*_) > 0, *m*_1_ is set as *m*_1_ = (*m*_1_ + *m*_L1_)/2, **M**_Up_ is updated as **M**_*i*_. In both cases, **M**_*i*_ is calculated as [0 *m*_1_ (1 − *m*_1_^2^)^1/2^].
- *Step 4* The newly-generated **M**_*i*_ from Step 3, together with the reference beam **I**_*i*_, are used to construct new refractive planes **N**_m1_ and **N**_m2_. Step 2 is then repeated with these new planes.
- *Step 5* Steps 2 to 4 are repeated iteratively to continuously adjust the refractive planes until the reference beam **I**_*i*_ passes through the center of the second refractive plane as closely as possible. Figure 10 shows the algorithm flow chart.

**Fig. 11:**
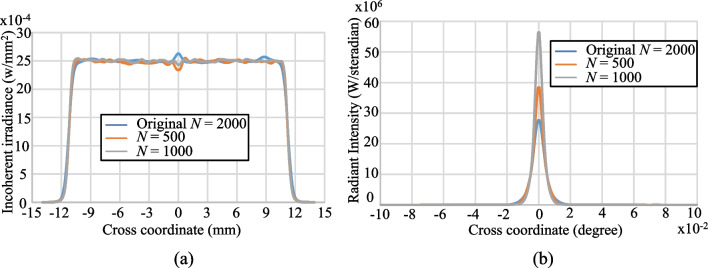
Simulation results for aspherical lens based on center of refractive planes: **a** cross-section of spot irradiance, and **b** cross-section of radiance intensity

Notably, the proposed algorithm yields a significant reduction in the number of refractive plane segments required to achieve the desired beam shaping performance. For example, Fig. 11a shows that only *N* = 500 segments are required to achieve the same irradiance uniformity as the original lens with *N* = 2000 segments. Furthermore, Fig. 11b shows that the proposed algorithm also provides a better collimation performance than the original method. The proposed algorithm running in common laptop is around one second to produce the polygon object file of the Zemax for *N* = 1000. The simulation results show that the power efficiency can be around 99% through the use of the aspherical lens designed by the proposed algorithm.

**Fig. 12:**
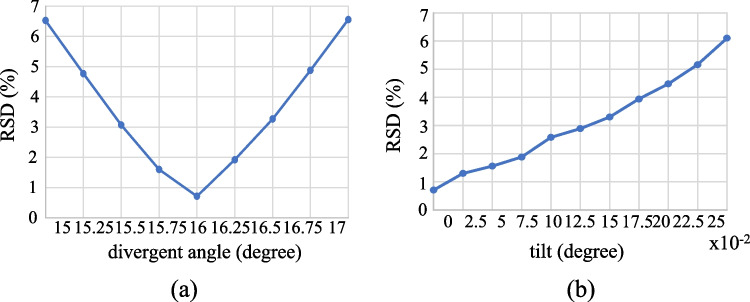
The investigation of the light source variation **a** divergence **b** tilt

The light source for the proposal can be of any distribution, but it must be rotationally symmetrical to simplify the design process. Since the Gaussian-like light source is easier obtained from the HeNe laser, the aspherical lens design is based on the Gaussian distribution for the demonstration. However, the light source could be unstable on the divergent angle and rotation-symmetrical requirement during experiment. The standard deviation (RSD) [26] is applied to investigate the light source variation on the divergence and tilt, that is,

12 $$RSD=\sqrt{\frac{1}{{N}_{P}}\sum_{i=1}^{{N}_{P}}{\left[\frac{{I}_{T}\left(i\right)-{I}_{0}(i)}{{I}_{0}(i)}\right]}^{2}}$$

where *N*_*P*_ is the number of the major contributed points, *I*_*T*_(*i*) is the simulated illuminance with tolerance at the *i*-th major contributed point and *I*_0_(*i*) is the illuminance at the *i*-th major contributed point with no tilt and divergent angle 16°. Under the RSD 5% requirement, the divergence variation allows within 0.75° but the tilt must be less than 0.2°, as shown in Fig. 12.

**Fig. 13:**
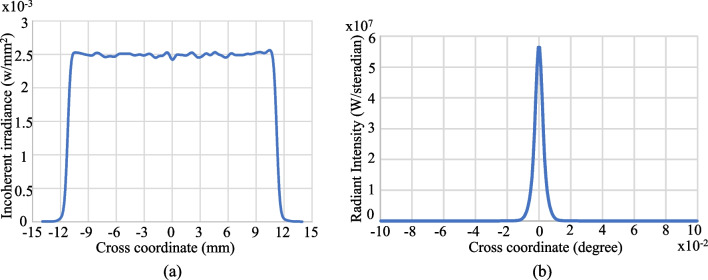
Simulation results for aspherical lens with *N* = 1000 from the light source size 3 × 3 mm^2^: **a** cross-sections of spot irradiance and **b** cross-sections of radiance intensity

The light source size could also affect the output beam profile. Figure 13 shows the simulation results by the light source size with 3 × 3 mm^2^. The results show that the uniformity and collimation keep great results. Current LED size can be developed less than 3 × 3 mm^2^. Even there are many micro LEDs developed for the display. Therefore, the proposal could be a way to employ common LED light source to design the aspherical lens for beam shaping system.

## Aspherical lens design with specific beam shaping profile

The main task of a beam shaping system is to generate a collimated uniform output beam from an originally Gaussian distribution light source through a designed lens. However, the semiconductor lasers commonly used as light sources nowadays have a divergent rather than Gaussian distribution. Moreover, the output light pattern is often required to be not only collimated, but also to have a specific intensity distribution. In order to obtain a rotationally symmetric collimated output beam with a particular irradiance pattern, the heights of the segments on the second surface of the aspherical lens can be obtained using the method previously described for the heights of the segments on the first aspherical surface in Sect. 2. For example, assuming that the desired output light profile is defined as *I*_output_(*y*), the heights of the refractive planes on the second aspherical surface can be calculated from Eq. (13) as

13 $$\begin{array}{ccc}\begin{array}{l}{P}_{2,i}={\int }_{{y}_{2,i}}^{{y}_{2,\left(i+1\right)}}{I}_{output}(y)ydy\\ =\frac{{\int }_{0}^{{R}_{2}}{I}_{output}(y)ydy}{N}\end{array}& \mathrm{for}& i = 1, 2, \dots , N\end{array},$$

where *R*_2_ is the radius of the output beam, and *y*_2,1_ = 0 is the initial *y*-coordinate value of the second aspherical surface. The heights *y*_2,(*i*+1)_ of the refractive planes on the second surface can be calculated using numerical methods.

The feasibility of the proposed method is demonstrated in the remainder of this paper for two specific collimated output light profiles, namely ring-shaped and triangle-shaped, defined respectively as

14a $$I_{{output}} \left( y \right) = \left\{ {\begin{array}{*{20}l} {2.57 \times 10^{{ - 3}} } \hfill & {for\;2 < y < 11.3} \hfill \\ 0 \hfill & {otherwise} \hfill \\ \end{array} } \right.$$

14b $${I}_{output}\left(y\right)=\left\{\begin{array}{ll}3.3\times {10}^{-4}\times y& for 2<y<11.3\\ 0& otherwise\end{array}\right.$$

**Fig. 14:**
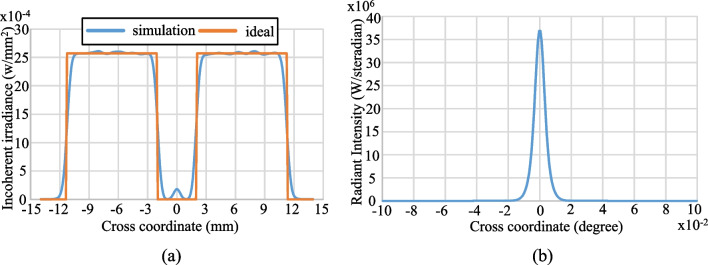
Ring profile simulation results: **a** incoherent irradiance, and **b** radiant intensity

**Fig. 15:**
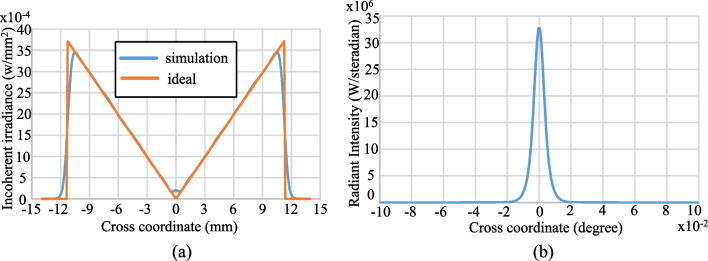
Triangular profile simulation results: **a** incoherent irradiance, and **b** radiant intensity

Figures 14 and 15 show the simulation results obtained for the irradiance patterns of the two aspherical lenses designed in accordance with the requirements of Eq. (14a, 14b). (Note that both lenses were designed using the algorithm described in Sect. 3 with *N* = 1000.) As shown in Fig. 14a, the simulated irradiance distribution closely resembles the ideal ring-shaped distribution. Moreover, the collimation error is less than 0.02° as shown in Fig. 14b. The results presented in Fig. 15 show that the designed lens also satisfies both the triangular light pattern distribution defined in Eq. (14b) and the collimation requirement.

**Fig. 16:**
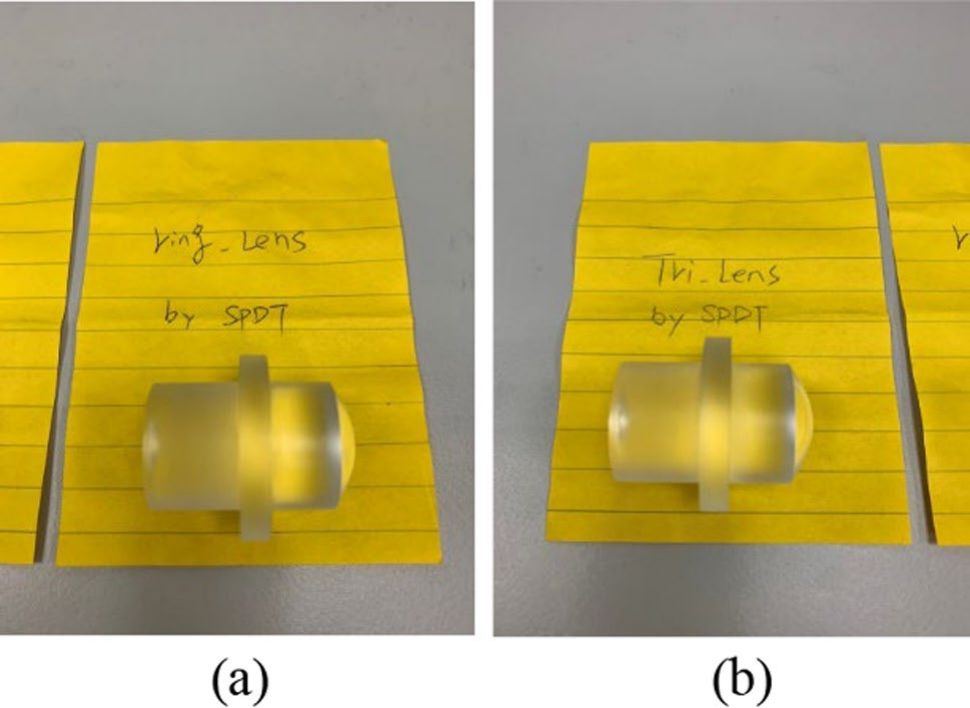
Photographs showing aspherical lenses designed to produce: **a** ring-like and **b** triangle-like intensity distributions

To validate the simulation results, two aspherical lenses were fabricated from PMMA as shown in Fig. 16a and b, respectively. The experimental aspherical lenses come from a contract manufacturing. Although the proposal focuses to develop the algorithm in the aspherical lens construction with specific collimated output profile, the segment points from the algorithm are applied to find out the coefficient of the aspherical polynomial for the aspherical lenses fabricated. Current technologies in the industry promise the tolerance with sag precision, tilt and decenter under 1 μm, 0.1° and 1 μm. It should be sufficient precision for the most segments of the aspherical lens.

**Fig. 17:**
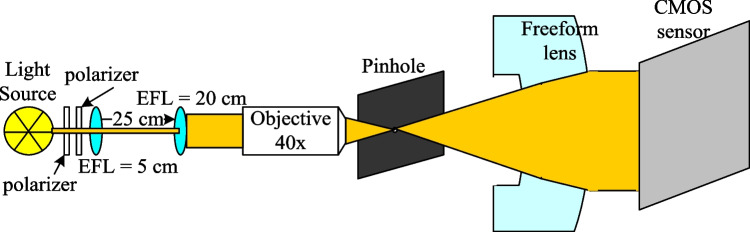
Experimental architecture

**Fig. 18:**
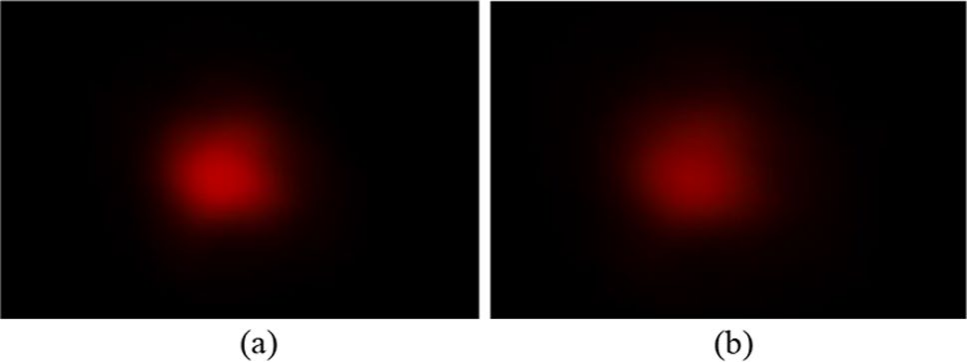
Spots on CMOS sensor located at different distances from pinhole: **a** 40 mm, and **b** 50 mm

**Fig. 19:**
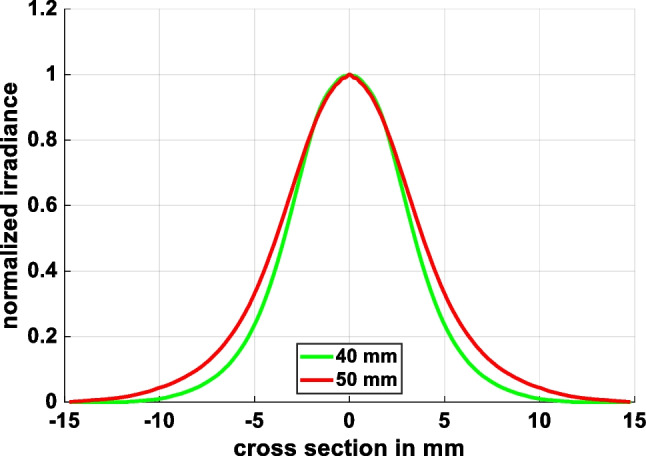
Spot cross-sections of divergent light source at distances of 40 mm and 50 mm from pinhole

Figure 17 shows the experimental setup used to evaluate the uniformity and collimation of the two output beams. A 633-nm HeNe laser was used as a collimated light source, with two polarizers placed in front to control the power level. The laser source was first expanded by two lenses with effective focal lengths of 5 cm and 20 cm, respectively, and then passed through a 40 × objective lens and a 10-µm pinhole to transform the collimated light source into a divergent light source. The resulting light source distributions (without the aspherical lenses inserted in the optical setup) were measured at distances of 40 mm and 50 mm behind the pinhole, as shown in Fig. 18a and b, respectively. Figure 19 compares the cross-sections of the two light spots at distances of 40 mm and 50 mm from the pinhole, respectively. It is observed that the divergence angle is around 16 degrees in both cases.

**Fig. 20:**
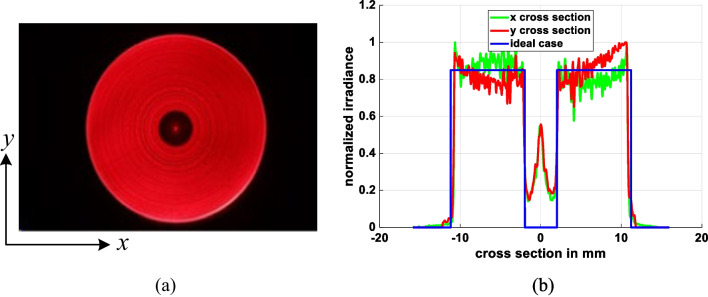
Experimental results for ring profile lens: **a** 2D spot irradiance, and **b** cross-section of radiance intensity

**Fig. 21:**
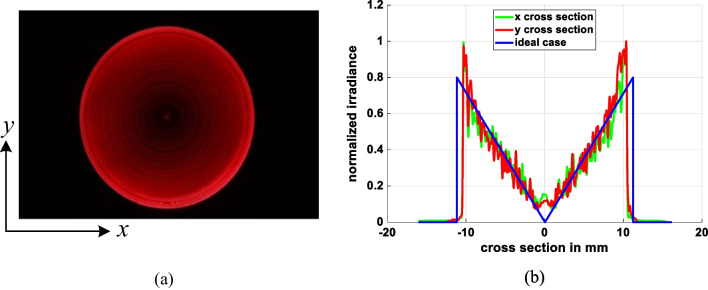
Experimental results for triangle profile lens: **a** 2D spot irradiance, and **b** cross-section of radiance intensity

**Fig. 22:**
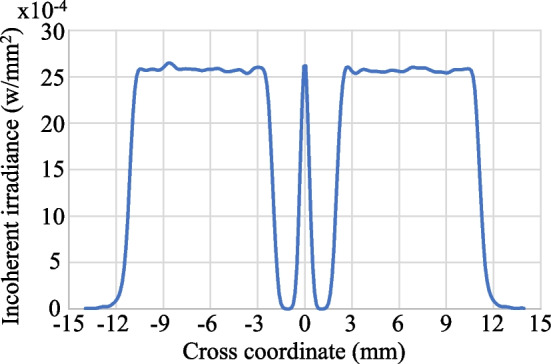
Ring profile simulation with *N* = 200 refractive segments

The beam shaping system often requires that the light source must be stable because aspherical lenses are based on its distribution during design process. However, the experiment light source has some problems like divergence variation and not rotation-symmetrical. By warming up the HeNe laser at least 30 min, we can obtain more stable light source with the divergent angle close to 16° and the distribution approach to rotation-symmetrical. In evaluating the performance of the fabricated aspherical lenses, each lens was placed 25 mm behind the pinhole, and the CMOS camera was positioned 100 mm behind the aspherical lens. Figures 20 and 21 show the output light patterns obtained from the lenses with a ring-shaped intensity profile and triangular intensity profile, respectively. The experimental results from the ring profile show that approximately 20% of the power is concentrated at the center. This is due to insufficient precision of the aspherical lens at its center. When we simulate the ring profile using *N* = 200 refractive segments, depicted in Fig. 22 the results indicate a similar effect to the experimental findings—specifically, a peak in power at the central part. However, it is still seen that the light spot conforms to the desired output light pattern in both cases.

## Conclusions

This study has proposed a simple method based on Snell’s law for the design of aspherical lenses capable of producing a collimated output light with a specific irradiance distribution in beam shaping systems with a rotationally symmetric divergent light source. The simulation results have confirmed the feasibility of the proposed method. However, the resulting aspherical lenses require a large number of segmented refractive planes. Thus, a further algorithm has been proposed which ensures that the reference beam passes through the central portion of the refractive plane and therefore reduces the total number of refractive surface segments required to achieve the required beam shaping performance. In the proposed algorithm, the related refractive planes of the two aspherical surfaces are updated iteratively until the solution is found which constrains the reference beam **I**_*i*_ to pass through the center of the refractive plane segments of the second surface. The validity of the design process has been demonstrated for two illustrative cases of a ring-like collimated light distribution and a triangle-like collimated light distribution, respectively. The experimental results have shown that the proposed method is indeed able to design aspherical lenses which transform a rotationally symmetric divergent light source into a collimated output light with a specific irradiance distribution.

## Data Availability

Data will be made available on request.
